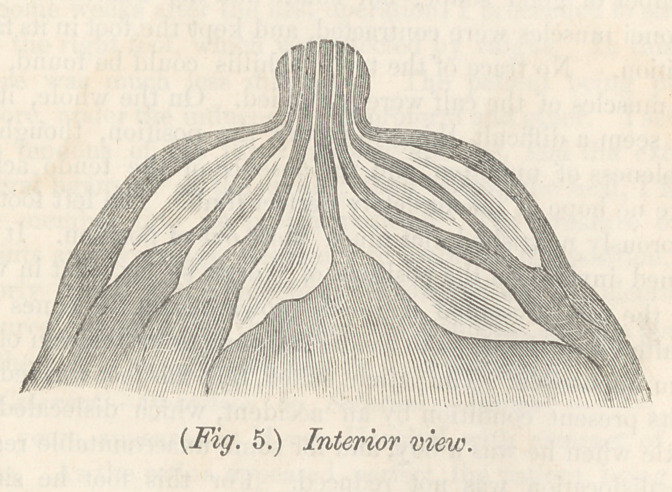# Inflammatory Affections of the Female Breasts

**Published:** 1860-09

**Authors:** W. H. Byford

**Affiliations:** Chicago. Professor of Obstetrics and Diseases of Women and Children in the Medical Department of Lind University


THE CHICAGO
MEDICAL EXAMINER

Vol. I. SEPTEMBER, 1860. No. 9

### ORIGINAL COMMUNICATIONS.

### INFLAMMATORY AFFECTIONS OF THE FEMALE BREASTS.

(Read to the Illinois State Medical Society.)

BY W. H. BYFORD, M. D., OF CHICAGO.
Professor of Obstetrics and Diseases of Women and Children in the Medical Department of Lind University.

Inflammation attacks the mamma of infants, children and youths of both sexes, women childless or senile may be the subject of this affection, but in the present paper I desire to confine myself to the subject as manifested in the pregnant, puerperal and lactiferious conditions of females, the conditions in which the mamma are functionally active or preparing for the discharge of their duty.

Inflammation may invade the tissue in and about the breasts—as

1st. The invoulcra—

(*a.*) The skin and integrements, areola glands, follicles &c.

(*b.*) The suspensory fascia covering and containing the whole breast, and its intra and sub-glandular processes and laminæ.

2d. The lymphatic glands, superficial and deep-seated.

Or the structures entering more intimately and essentially into the formation of these—as

3d. The nipple and milk ducts contained within it, constituting the eliminatory apparatus.

4th. The sub-areolar expansion of the milk tubes, called by Sir. Astley Cooper, milk reservoirs.

These reservoirs actually occupy nearly the whole front part of the breast, immediately beneath the integuments and fascia, and lie above the gland in all parts of the breast except the margin, where the hard substance of the gland may be left.

5th. The gland and cellular tissue which pervades every part of it, and is the medium of connection between the lobes, lobules, tubes, vessels and nerves of its substance.

Although very few cases of mammary inflammation occur in which the disease is confined to one structure, and many in which several are simultaneously invaded, yet I think an intelligent anatomical division will conduce to clearer views on the subject. I shall therefore base what I have to write about mammary inflammation upon the foregoing consideration with respect to its seat.

It might be supposed that the integuments or involucra of the breasts were as liable to disease at one time as at another, and hence, at the time when the various processes connected with generation are passing should enjoy their usual exemption from disease, but experience proves the contrary.

It may not be expected that I shall dwell to any length upon the eruptive or specific diseases which may attack the breast, for they may occur at any time, nor erysipelatous and rheumatic affections which more frequently than is generally believed attack the structures. Rheumatism of the fascia of the breasts I think I have witnessed repeatedly. It is manifested by the usual characteristics of it when other parts are attacked. Almost the only sort of inflammation to which the integuments are subject that can particularly interest us now is phlegmonous. Of course the real seat of the inflammation, or at least its beginings is in the arealar tissue beneath the skin. Generally it is circumscribed and single in locality, often there are several simultaneous or successive foci, less frequently it is quite diffuse, involving a large surface, causing great deformity and damage to the organ, and attended with serious constitutional disturbance.

Phlesmonous subcutaneous inflammation in the breasts is attended with the symptoms which usually accompany it elsewhere, pain, heat, redness, swelling, hardness, tenderness in the early stages, varying in intensity with the extent and acuteness of the affection. We may generally diagnosticate this from inflammation in other tissues of the breast by isolation. There is usually no trouble in the secreting, eliminating or containing apparatus of the breast. The functions of the whole organ are properly discharged. The inflammation is one generally of inconvenience instead of damage. It is superficial and we may ordinarily get below it so that we can assure ourselves it is outside the mammæ. Most frequently the areola is the seat of this disease. There can be no question, however, but that the deep cellular tissue is as often the subject of inflammation as any other of the deep structures, and indeed some good pathologists think it is the seat of disease when we suppose the gland to be the part affected. However this may be, inflammation of any of the deep tissues, generally brings this into the morbid mass. Inflammation of the superficial areolar tissues occasionally involves the reservoirs or glands by contiguity. I have but little doubt that the diffuse inter-mammary suppuration which we see sometimes take place and produce such prodigious quantities of pus, often melts down the connective areolar tissue distributed between the lobes, lobules, and tubes of the organ without always at least attacking the more essential structures. I cannot hope however, nor do I design to try to distinguish between deep-seated cellular and glandular inflammation. The distinction, if made, so far as I can see, would not lead to any favorable result. Chronic superficial cellular inflammation does not often occur, except as it becomes chronic by a long continued succession of small abscesses. It is possible also that the chronic sequela of cellular inflammation as exibited in hard tumors may be of this character in some instances. When this is the case we should expect to find the hardness not so defined, but shaded off into other parts, somewhat regular in outline, and not sharp irregular and lobulated.

---

*Inflammation of the Nipple.*

This may be accompanied with abrasions, fissures or ulceration. Abrasion is most frequently seen on the apex of the nipple, and is the condition in which the delicate epidermis is removed by action of the child's organs in sucking, leaving the dermis naked, bleeding and raw. It may, however, be observed on any part of the nipple. Not unfrequently these abrasions are increased in depth by ulceration, until a greater or less portion of the nipple may be destroyed. Cracks or fissures likewise often affect the nipple. These cracks are located either on the top, sides, or at the base, of the organ. The apex of the organ, sometimes, is so deeply fissured, as to lay it open to the bottom of this projection, and leave it split in halves; but usually much less extensive, and it simply lays open the top of the nipple to the depth of the skin. The worst fissures that occur on the nipple, however, generally more or less completely encircle the base of the organ. To such an extent are fissures of the base carried by ulceration, sometimes, as completely to amputate this little projection. Abrasions and fissures lead almost invariably to ulceration, and we may consider these as the first stage, so to speak, of ulceration.

This ulceration, of course, resulting as it usually does from abrasions and fissures, occupies the place which I have assigned to them.

The symptoms which accompany these three conditions of the skin of the nipple, do not differ each from the other, and without inspection, we would not probably be able to distinguish between them. There is great pain upon handling the part, or when the child sucks; indeed it is so very severe, as to render it entirely intolerable to the patient, and cause her to resist every request, or even command, to nurse the child. When the child is put to the breast, in addition to the pain, they bleed so as to disorder the milk, and sometimes sicken the child and cause it to vomit up the contents of the stomach. The extent to which ulceration may proceed under the irritating influence of nursing, is sometimes very great.

I remember an instance in a patient affected with stomatitis materna, when the nipple was completely destroyed, and the place where the nipple had been, excavated below the surface before ulceration was arrested. Every experienced physician must have seen cases where the nipple was cleft, cut off, or very badly mutilated. Ulceration has its origin in many cases, also in small phlegmonous inflammations of the cellular tissue of the nipple. It not unfrequently happens that small pimples arise, suppurate, burst, and on account of the constant irritation of nursing, remain open and pass into a state of ulceration, which is often very obstinate.

Small ulcerations occur in the same way on the areola occasionally, but not with any thing like the frequency, as in the nipple. Neither are they generally so painful as when upon the nipple. The parts being less firm, the swelling does not press upon and distress the surrounding parts so greatly. Such diseases of the areola get well much easier than those upon the nipple, because they are less disturbed than in that place by the child when sucking.

---

*Inflammation of the Lymphatic Glands of the Mamma.*

It is important, in a diagnostic point of view, to bear in mind the frequency of inflammation of these glands. As in other parts of the body, so in the breasts; they inflame in consequence of the passage of acrid or unhealthy lymph through them, derived from inflamed tissues. Ulcerations and abrasions of the nipple and areola are frequently followed or accompanied by the inflammation of these bodies. No doubt enlargement by deposit, leading to chronic inflammation, may also sometimes occur, independent of inflammation. The indolent tumors over the gland and near its margin on the inner, outer and upper circumferences, are frequently chronically inflamed lymphatic glands.

The *symptoms* of inflammation of these glands do not differ in the acute form, from those attendant upon superficial phlegmon. All the distinguishing circumstances of inflammation are experienced. They are more circumscribed than ordinary, the margin is more defined and does not shade off into the healthy tissues, but appears, as it were, encysted. This is the case, however, only at first, as the inflammation often, in fact, I think generally, spreads to surrounding tissues, when the difference cannot be clearly made out.

As the inflammation subsides, there is left for much longer time, hardness, than in phlegmon of the integuments. The acute symptoms merge into chronic, and hardness, tenderness, and in many instances discoloration, last a considerable time. Suppuration does not occur so quickly as in phlegmon, and resolution much oftener. To make out a diagonosis, we should remember the most common seat of the two. They are ordinarily both (phlegmon and inflammation of the lymphatic glands) small in size, usually not larger than an English Walnut; but phlegmon occurs about the areola, while the other is usually over the located gland, and near its margin. The phlegmon may occur in any direction from the nipple with reference to circumference, but lymphatic inflammation is situated at the inner, or outer-upper edge of the mamma. In scrofulous or broken down patients, a chronic condition of inflammation is likely to take possession of these glands, or they may be filled with albuminous accretions and undergo indolent changes, which might lead the inexperienced to fear malignant disease. I have a patient which has a deep lymphatic tumor in the breast on the axillary margin, who assures me it has been in the same condition for six years. This tumor is hard, round, regular, a little flattish, freely movable, and resembles lymphatic enlargements at the clavicle and groin, in the same patient.

They may be usually distinguished from malignant tumors when indolent and not tender from inflammation, (for malignant tumors are not sensative in the beginning,) by being more rotoundly irregular, without the sharp outline generally characterizing malignant disease. If they are livid, they are also tender; if they involve the skin, they are tender to the touch, and the skin is inflamed, neither of these conditions obtain in malignant cases. The malignant tumors may be livid and almost insensible, it may involve the skin, attach itself to it, and not inflame it.

The lymphatic tumor is hard alike all over; if softer in one part, that part is the center. The malignant is harder in the center until nearly ready to ulcerate. When the lymphatic tumor has ulcerated, the cavity is regular, and red or pale about the edges, and secretes pus. The malignant ulcer is ragged and exceedingly irregular, in fact, sharp irregularities of edge and cavity mark peculiarly malignant ulcerations—the edges are livid, not red or pale, ichor instead of pus is produced. In the ulcerated lymphatic there is no smell ordinarily, certainly none but the smell which may arise from uncleanliness; a malignant ulcer will smell in spite of us (!) and the smell is peculiar, and when once noticed, will be recognised without difficulty again. Lymphatic glands may be inflamed singly or in numbers, several being the subjects of inflammation at the same time, or only one. As I have before intimated, the disease may be chronic or acute, (indolent or active.)

---

### *Milk Abscess.*

Passing to the deeper structures of the breast, we encounter inflammation of the containing portion of the mamma, the expanded milk tubes, the milk reservoirs. There are from fifteen to twenty-five of these expanded tubes, holding from two drachms to an ounce each, in the natural condition.—They are separate and distinct, each tube representing a lobe of the gland. One or more of these may inflame, ulcerate and discharge the milk, mixed with greater or less quantity of pus. Inflammation, followed by ulceration and discharge of pus and milk of these reservoirs, is alone what should be called milk abscess. Abscesses from this part of the breast do not occur singly, as a general thing; several are going on at the same time, one arriving at the ulcereted stage after another: so that we have a succession, each abscess involving one tube, and sometimes, but not often, more. They are seated under the anterior surface of the breast, mostly within an inch of the areola, and sometimes under it. In some persons the reservoirs are large, extend a considerable distance in every direction from the areola, and overlay the gland almost to the margin of the mamma. Milk abscess need not necessarily be near the central portion of the organ, although they generally are not far from the areola. They usually proceed somewhat slowly, taking longer to arrive at the suppurative stage than in superficial phlegmon. Swelling and tenderness are felt near the areola, it increases steadily until an apex is observed in the tumor, the integuments are thinned, fluctuation is observed, and rupture follows. This process requires a very different length of time under different circumstances. If the milk is secreted rapidly, the tube is distended faster; if secretion is scanty, the advance is slower. The inflammation depends upon distension of the reservoirs by milk which cannot find its way out of the milk tubes. Retention of milk is caused by several different circumstances, which I shall have occassion to mention after awhile. I wish now to be understood as saying, that it is the essential cause of the inflammation in this form of disease. The milk is secreted, but not eliminated, from the reservoir affected; it acts as an irritant by its great accumnlation, until inflammation is the result. The secreting capacity of the organ is not necessarily disturbed, and the excretion of the milk may be ready and easy through all of the tubes whose reservoirs are not affected, and we may think it is being evacuated entirely, while it is retained in one or more reservoirs by the stoppage of the nipple tubes. When evacuation, either spontaneously or by the lancet, is affected, pus and milk flow in moderate quantities at first. The pus gradually diminishes, the milk becomes more pure, until a milk fistula occurs, which lasts a greater or less length of time. Should the eliminative tube become open, and allow the milk to flow from the affected reservoir through the nipple, the adventitions opening may entirely heal, and the integrity of the part be restored; but as is most frequently the case, the fistula remains open, until the breast ceases to secrete, all the milk produced by the lobe, whence the reservoir is supplied, flowing out at the place.

Sometimes, again, after breaking and discharging, it suddenly heals up, distension recurs, and the process of ulceration and discharge is repeated.

The sympathetic symptoms are not generally so great as in some other varieties of mammary inflammations. Fever does not run so high, aching of the head, limbs, &c., do not distress the patient so much. Yet they sometimes are quite considerable, and require alleviation by appropriate remedies. The damage done to the breast by inflammation attacking these parts, is not so great as results from glandular inflammation generally, though I have known instances in which nearly all the reservoirs were destroyed, and the breast henceforth reremained useless. One of the worst features of the case is derived from the persistent repitition of abscesses, wearing out the patience of the medical attendant, and the powers of endurance of the patient. It is always complicated by disease or deficiency of the nipple. Besides this ulceration or phlegmonous inflammation of the milk reservoirs, there is another form in which blood and pus are discharged through the nipple tubes, the passage from them being free. Very few experienced physicians but what have seen this discharge of pus, blood and mucus, from the milk tubes, with tenderness and some tumefaction under the areola. It is generally considered to be an abscess discharging in this way, but it is ordinary inflammation of the lining membrane of the milk reservoirs discharging its products through the nipple. Abscesses occuring as the effect of over-distension of the reservoirs, do not give origin to those deep ungovernable sinuses that sometimes trouble us in glandular inflammation, and while there is often milk fistula following them, these close as soon as the secretion ceases, and we have no further trouble.

Several times in my life I have met with these abscesses during pregnancy, in which the accumulation of pus and milk was very great, so that when they are opened, many ounces of pus and imperfectly formed milk were discharged. Several months since I was called in consultation in a case in which the disease had began three months before labor, and when I saw her the child was two months old, and large collections of pus and milk existed, pent up in the reservoirs of impermeable tubes in both breasts, and while some of the reservoirs contained, and their tubes discharged milk, upon nursing, half of them were the subjects of perulent inflammation. Generally the inflammation which causes the evacuation of the milk and pus, checks the secretions of milk, and the patient recovers before the time for labor. This is fortunate when it occurs.—According to my experience, this is the most common of mammary abscesses; indeed I think by a large majority.

---

*Glandular Abscesses of the Mamma.*

This is the most grave of acute inflammation of the breasts occuring during lactation. I am not aware of ever having seen an instance of mastitis proper, unless caused by violence in any other than nursing women. When the inflammation takes place early in nursing, it usually comes on about the third or fourth day. Mastitis cannot in the first few hours, be distinguished from the intense congestion which occurs at the time the secretion of the milk is first produced. In either case the woman is seized with a severe chill, in which it is not uncommon for her to shake and chatter as in violent ague. In the course of an hour, or sometimes longer, sometimes in a few minutes, the chill gives place to a violent reaction ; a high fever, pain in the head, limbs, back, and often abdomen, annoy the patient. All the phenomena of severe inflammatory fever occur. When the congestion subsides into a copious effusion of milk in the cells of the gland, the fever declines, a copious perspiration appears over the whole surface, and comfort succeeds great uneasiness, and sometimes alarm.

When, however, the gland is not completely relieved by secretion, this transition from a state of febrile reaction is imperfect and the patient left with more or less of the symptoms of fever.

Simultaneous with these general symptoms there is pain, tumefaction, tension, heat and tenderness of the mamma. If the secretion is established, the breast, as the sweating stage advances, becomes soft, cool and less sensitive, until it is entirely comfortable. On the other hand, if inflammation is to succeed this congestion, some part of the organ is left in a hard tender condition. A hard lump of greater or less size continues to occupy some deep portion of the breast. Tenderness, tumefaction, heat and redness increase until imflammation is permanently fixed. Without early, energetic and appropriate treatment, the woman will lose part of the mammary gland by destructive suppuration.

In the beginning of glandular inflammation, if the part be attentively examined, the shape and position of the lump will enable us to determine the seat. It will be either deep in the central portion of the breast or in the marginal region. The tunical part is irregularly lobular depressions and elevations may be observed, nodulus, not sharp ridges. Very soon after the inflammation begins, particularly should it be advancing, this nodular feel is merged in diffuse hardness of the surrounding parts, until the whole tumor may become smoothe and irregularly defined. Inflammation, hardness and tenderness increase for a few days, when the centre becomes slightly soft at first, growing more so until distinct fluctuation is perceived.

At this time we find a soft fluctuating locality completely margined by hardness all round. This then will be the feeling of a mammary abcess, whether acute or chronic. Glandular abcess differs from milk abcess, by being at first much deeper, having a covering of integuments &c., half an inch or more in thickness, while the milk abcess though quite hard, seems to be immediately beneath the integuments. When fluctuation is *first* perceptible in milk abcess it is shallow ; in mastitis it is deep and makes its way slowly to the surface. When pus arrives at the surface, and ulcerates through or is evacuated by the lancet, its flow is much more difficult and the evacuation less complete, relief is not so sudden and perfect. Extensive destruction takes placc both in the internal portions of the organ and in the integuments. And so tortuous and irregular are the tracks of transit, in some instances toward the skin, that the pus finds its way out with so much difficulty that the sinuses are sometimes extremely difficult to heal. This state of things may last for many weeks and even months. We not unfrequently find cases in which these sinuses are numerous, tortous and lengthly so as almost to riddle the internal of the organ, and discharge large quantities of pus, thus draining the system of the woman, inducing hectic, exhaustion and in extreme cases, death.

Often instead of begining at the time of puerperal congestion of the mamma, mastitits shows itself late in lactation. When occuring at such times, it may spring up suddenly, inducing all the general phenomena above described, in a greater or less degree of intensity, or it may be slowly established, and not bring the system into so decided sympathy and perturbation. Yet in the latter case, as the inflammation becomes more completely established, fever is pretty certain to be manifested, its intensity being greater or less according to the extent of tissue involved, the rapidity with which it advances, and the susceptibility of the patient. The first thing noticed, perhaps, is what the woman would call cake in the breast, of moderate, yet decided tenderness.

This consists in inflammation in one or more lobes of the mammary glands. It gets worse, the swelling becomes greater, tenderness more considerable, instead of the well defined nodulor tumor, the swelling is more diffuse—other parts are involved—the areolar tissue around the gland—redness in the skin is observed, sympathetic fever sets in, and then it passes through the different grades above mentioned in the acute variety, with less intensity.

Again chronic glandular inflammation is occasionally observed. At first a deep-seated suspicious hardness is felt in the breast, with barely tenderness enough to make the woman careful about hard pressure. It is usually well defined, nodulated in shape, moveable, and the parts free from morbid color or heat. It may be of small size, only involving one lobe of the gland, or a large part of the breast. In the beginning the distinctive mark of chronic inflammation, is tenderness to decided pressure, when first perceived. As the disease advances, it may of course be recognized by more and more decided symptoms. They are not unlike those I mentioned in connection with disease of the lymphatic glands of the breast. While in very many instances the inflammation of the different parts of the mamma occur separately and may be easily distinguished, we often meet with cases in which the different parts are simultaneously or consecutively involved. Something like the following order of things may take place: Abrasions of the nipple from the act of suckling runs into ulceration, milk abscess succeeds, bursts and heals, or not; mastitits or inflammation of the gland comes next, suppuration from the deep tissues, &c. The long continuance of mammillitis is very likely to be followed by inflammation of the milk reservoirs, and when these last continue, the seat of disease for any length of time, we may look for disease of the gland. There is one or two other points with respect to diagnosis between milk abscess and glandular, that I deem it best to speak of here. When the reservoirs are the seat of abscess, the milk is retained partially, or wholly, and is evacuated with pus when the abscess is opened.

In glandular inflammation milk is suppressed more or less perfectly, owing to the amount of tissue involved.

---

*Causes of Mammary Inflammation.*

As I have intimated, the pregnant puerperal and suckling conditions of women may be regarded as predispositions to mammary abcess. Women are much more liable to them when in these conditions than at any other time. Hence it would not be improper to say that these states of the system are predisposing causes of mastitis and its associate inflammation. The physiological congestion preceding and accompanying the commencement of lactation, very frequently is carried too far, and merges into pathological congestion, and this again into inflammation. When inflammation arises from this cause, it will almost invariably be mastitis or glandular inflammation. This sort of congestion may occur later, but usually it is in the puerperal condition. Another sort of congestion which often runs into inflammation of the glands is brought about by sexual intercourse in very excitable nursing women. I think I have known several instances of this kind. Other passions as anger, may be succeeded by like results. Vascular excitement from stimulants will endanger the breasts in puerperal women also. External causes may give origin to similar sorts of inflammation, as bruises from blows, tight lacing, stays of wealebone, &c. These last (45) are productive of a good many cases. Not unfrequently our patient gets up well from the effects of labor, and the first time she dresses to go out, pinches her excitable gland with lace strings, or punches it with the end of a piece of whalebone during the whole of her round of fashionable calls, and comes home with the breast excited to inflammation. Cold, acting partially upon the person, as the feet, the breast themselves, or even upon the general surface, repels the blood to the already blood-loaded gland, produces congestion as the first step of inflammation. Other external causes operate upon the nipple and surface of the breast, irritate the skin or destroy its integrity, &c. The child often sucks off the epidermis, and by thus abrading the nipple, ulceration is brought about.

Allowing milk or saliva to remain in contact with the delicate skin of the nipple or areola long enough to undergo decomposition, too often is the cause of ulceration, more especially when the saliva of the child is rendered poisonous by the existence of aphthous incrustation upon the tongue, gums, and roof of the mouth. The cracks so often found upon the nipples, I think is almost invariably produced by the habit of allowing the fluids deposited upon the delicate skin to slowly evaporate, and thus carry off, or otherwise neutralize the sebaceous unction of these parts, which is intended to keep the cuticle pliant and soft.

There is a class of causes which I am disposed to call pathological, very prolific of grave mammary diseases. One affection may act in producing another. Thus, ulceration of the nipple prevents proper efforts to draw the milk from the reservoirs; they become distended to a degree that causes inflammation, or the ulceration on the top of the nipple, by the swelling it causes in the inter-tubular tissue, lessens the diameter of the tubes, or entirely closes up their mouths, so that milk cannot find its way out or be drawn, accumulation results, and inflammation follows. Cracks, of course, will do the same; or, again, the inflammation originating on the nipple, may creep down the lining membrane of the milk tubes into the reservoirs, or even farther, through the ramification of the radicles of these ducts, to the substance of the gland itself. In either of these localities, suppurative inflammation may arise, and proceed through all its most aggravated forms. Contiguity of imflamed parts, may awaken inflammation in other parts. Integumentary inflammation, may extend to the reservoirs or glands, by spreading from one tissue to another. There can be but little doubt that acute, and in most cases, chronic inflammation of the lymphatic glands, is generally secondary to inflammation and ulceration of the nipple and areola. It would probably be too strong an assertion to make, to say that inflammation of the lymphatic glands, always has its origin in this way; for in cases of strong predisposition to this disease—and there are numerous instances of that kind—it would probably arise without much cause of excitement. Certainly, I cannot be mistaken in supposing that I have seen several such cases.

Anatomical causes of inflammation of the breast exist to a great extent. They are sometimes congenital and hereditary, but I think for the most part brought about by improper dressing. The flat, undeveloped, or retarded nipple, is one form of anatomical peculiarity which prevents the perfect performance of suckling, as is represented in figure 1, in the plate. The retention of milk will lead to milk abcess. Nursing is impracticable in this breast. Fig. 2 represents a breast with a very broad but extremely short nipple, entirely too large for a child's mouth, and so short, as to add to the difficulty of prehension. Fig. 3 represents a breast with scarcely a trace of the peculiar warty tissue, like nipple, and is simply pouched slightly where the nipple ought to be. A very small nipple, where the milk tubes seem to be bound in such a contracted bundle, as not to allow free egress to the milk, is represented in figure 4.—These four specimens of nipples which we often meet with, are almost impracticable. The first and third, quite so; and the second and fourth, so difficult, that we are generally driven to the necessity of abandoning it, after the best directed efforts to make the breast available. The danger to breasts furnished with such nipples, is that the milk will not be properly evacuated, and that milk abcess will result. In fig. 5 we have a nipple large enough to be easily apprehended, and drawn by the child, but it is too constricted at the base. The milk tubes upon entering it, turn too acute an angle, a little swelling of the sub-areloar tissue from retention of the milk, will stop them entirely up, so that the milk will not pass out. In order the better to illustrate what I meam, I add a sectional view of this kind of breast and nipple. At *a* the milk reservories may be seen contracting at the nipple, forming the milk tubes, which turns abruptly upward, and even a little outward. This will be made still plainer by giving what I call a model breast and nipple, fig. 6. It speeks for itself. The nipple is slightly coin cal, the base being larger than the apex. I add also a sectional view of this breast. As will be seen, the milk tubes are free from pressure every where. Their entrance into the nipple, is by a slight`curve instead of angnlar turn. The milk will flow spontaneously from this kind of breast, and there can be no accumulation in the reservoirs. In nipples represented by fig. 5, the danger is, that milk, saliva and mucus, will collect in the groove around the base, decompose, and thus induce mammillitis with its attendents and consequences.—This could not well occur in the case of fig. 6. There is no lodging place, the nipple would be wiped clean of all these accumulations by the mouth, and return of the breast inside the clothing of the mother. The shape of the mamma may predispose it to disease, but not in so striking a manner. The more conical a breast the better. A flat sessile mamma is more likely to inflame. Although the above mentioned varieties of nipples are not the only ones predisposing to mammary abcess, yet by drawing the attention of the profession to to the subject, thus distinctly, it is believed there will be no difficulty in recognizing adverse anatomical peculiarities whenever they do occur.

It might be appropriate to examine into the cause of these anatomical differences, in the shape of this interesting organ, but the length of my paper will not allow me to indulge in this direction. Like all other formations, the nipple would doubtless differ under the same circumstances in different persons, naturally, but I think there is no doubt, much of the deficiency is produced by tight lacing, and the pressure made directly upon the nipple, for a series of years during its development.

More regard in dressing, as well as education, is bestowed upon fitting the young lady to get married, than to perform her functions properly, after getting married.

---

*Treatment.*

I can better give my views of the treatment of the affections above described, by observing the same general division with reference to the application of the processes of cure. Inflammation of the nipple will come up in this order of the arrangements for consideration, first. Our means of cure for mammillitis should be arranged under three different heads, as follows: Prophylactic, palliative and curative. The first have for their object the preparation of the nipple for the trials through which it has to pass, at the time of nursing. As has been seen, the causes operating upon it produce abrasions or chaps, and their action is greatly facilitated by the natural and acquired tenderness of the structure, particularly the epidermis and skin. The prophylactic means to be used are such as harden these. As elsewhere, so in the nipple, the skin becomes tough and the epidermic scales abundant and adherent, upon exposure to air and friction. The contrary condition will obtain—tenderness, &c.,—from pressure and covering, with impermeable or large quantities of goods. In this condition it is protected by extraneous covering, and hence does not furnish its own proper defence. The epidermis will be thin and light, and the skin tender. The nipple, therefore, should be covered lightly during pregnancy and nursing. The thinner and more permeable the covering, the better. It should be of such a character as freely to admit the air. At the same time, it should be subjected pretty constantly to moderately rough friction.

An excellent dressing for the nipple for the last two months, is a rough coarse sponge, so cut as to cover the areola, surround and cover loosely, but touch every part of the nipple. Over this there should be but one thin thickness of goods, so as to allow of the evaporation of fluids as fast as secreted, and the free admission of atmospheric air. In cold weather, when going out, the breast of course would be covered by all the over-clothing that are used for the protection of other portions of the person. It is a great mistake to cover these important organs—important on account of their usefulness instead of their beauty—so thickly as they usually are; they bear exposure with great impunity. When we wish to harden the nipples, we should bear in mind the circumstances which harden our hands, and make use of them; we should equally avoid the circumstances that soften our hands. When a lady wishes to soften and whiten her pretty little hands, she wears kid gloves, and does not allow them to touch hard substances. In a like manner she may soften her nipples, if she should wish to do so. To occasionally moisten them with water and allow it to evaporate slowly on exposure to air, is a good expedient; friction with a dry towel or the fingers, will assist in the process of hardening. It is a matter of great question, whether the various washes used to harden the nipples, are not injurious instead of beneficial. They generally exert a chemical as well as physiological effect, while this last is all that is desired. During lactation, the same exposure to air and lightness of covering should be observed, and after nursing, the nipple should be wiped clean and dry before being returned under the clothing. This is a rule that should never be neglected. Those who have observed the effect of allowing the udder of a cow to dry spontaneously after the calf is taken from her, will understand the importance of attending to this matter. It will be all the better to use a little glycerine or very fine olive oil after they are dried each time, particularly if we have reason to apprehend danger of chaps or cracks. Such prophylactic measures will very generally enable us to avoid the occurence of distressing chaps or cracks. When, however, the nipple becomes inflamed, these are not sufficient to satisfy the demands of the case, and we must resort to palliative and curative measures, and first of the palliative. As the nipple must be used in order to preserve the function of the breast, and as every time the child sucks, the healing processes that have begun must be more or less interrupted, it becomes important to procure such means as will preserve the breast from the effect of these interruptions as much as possible. The chaps and abrasions that occur, and give rise to inflammation and ulceration, may be located anywhere upon the nipple, at its summit, sides or base, and when the child nurses, the tongue and labia embrace it so closely, that none of these places escape. The artificial means used to palliate the effect of sucking, intervene between the mouth of the child and the nipple, and should be selected with special reference to each case. The shield of ivory or brittania answers very well when properly managed. They are made in the form of a conical hat, having a rim, a crown cavity, with a draught tube rising out of the top for the milk to flow through. Now, having in mind that these three parts must vary in length and size for different shaped nipples, and cases in which the locality of the abrasions or chaps are different, we will have no trouble in making a profitable selection. The rim should be large enough to cover the areola, the crown or nipple cavity large enough to pass over the nipple, merely touching it on the sides. These things should be observed in all cases. The depth of the nipple cavity is a matter of the greatest importance. If the abrasions or chaps are on the summit of the nipple, it should be so deep, that when drawn, the top of the organ will not touch, or else it will cause pain. There should be no pressure on the top. But if the cracks or abrasions are on the sides, or at the base of the nipple, then the cavity of the shield must be shallow, so that the top of the nipple touches its bottom in such manner as to prevent stretching the organ, and bring the pressure on the top altogether. In this latter case, the bottom of the cavity should be smooth as possible, and correspond in shape to the summit of the nipple, in order to prevent unequal pressure. The shield, of proper shape, size, &c., will afford great relief to the patient, and prevent very much the disturbance to the healing nipple. It is not a matter of indifference either, what material we use as an envelope for the shield.—Gum elastic or cow tets, are always clumsy, and easily become foul or hard, and sometimes taste in spite of our best efforts. Now, I cannot avoid the conviction that a soft linen rag properly adjusted over the draught tube, is better and cleaner than any other envelope. It has the advantage of being cheap and always at hand so abundant, that it may be replaced by a new one after each operation of sucking.

But a very ingenious contrivance is mentioned by M. Legroux, which I will describe:

| | | |
|---|---|---|
| ℞. Collodion, | ppts. | xxx. |
| Ol. Ricini, | " | ss. |
| Ol. Terebinth, | " | iss. |

Mix. This is a fluid mixture which is quite adhesive, and dries less quickly than collodion. It is applied upon the areola with a brush, so as to encircle—but not touch the nipple—the width of an inch. While yet soft, the nipple is covered by gold beater's skin, and pressed well down around it upon the mixture. The skin adheres to the adhesive material, and thus forms perfect, smooth and pliant covering to the nipple. All that remains to finish, is to prick several holes through the gold beater's skin with a needle, to let the milk through. This has the advantage of not changing the shape, size and feel of the nipple to the mouth of the child, so that it sucks more readily than it would an artificial nipple made with a common shield. But while this is the case, it allows the pressure of the lips upon the nipple at every point, and only partially relieves the mother from the pain.

In the most of cases, I would rely more upon the judicious selection and management of a shield, than this contrivance, ingenious and neat as it is. This may be imitated by other adhesive mixtures and tissues. Before sucking, the gold beater's skin must be moistened with a little sugar and milk. Much of the suffering under nursing, while the nipple is raw from chaps, abrasions or ulcerations, may be avoided by being drawn by the mouth of an adult, so shaping the vacuity produced for the purpose of drawing, as not to touch the sore part. If the lips are so placed around the nipple as to press upon the areola, and not touch the nipple more than very gently—and I am sure this is practicable by any intelligent adult who will make a persevering trial—the draught can be accomplished with comparatively little pain. Violent action should not be used, a gentle but constant pressure with the lips on the areola, with persevering but very gentle draught, will usually suffice, and powerful suction is sure to aggravate the cause of the retention of milk. I have often sat down, and by encircling the nipple with my fingers without touching it, and pressing upon the areola, caused the milk to flow freely, when with great difficulty it could be drawn out. In thinking upon this subject, we should remember that it is the pressure of the atmosphere upon the outside of the breast, combined with the elasticity of the integuments and coats of the milk reservoirs, that urges the milk forward through the nipple into the vacuum caused by excluding it from around the top of the nipple. The vacuum will not be necessary, if the pressure can be made with sufficient firmness without injury of the part. Why may not some ingenious individual invent a milking apparatus of gum elastic, that by pressing upon the areola and front of the breast, without causing a vacuum on the nipple? This would often save a great deal of trouble and suffering to our lady patients. In thus viewing and treating the subject, we would push the milk out, instead of, as we upon a superficial look at the matter, suppose, pull it out.

The above palliative means do not enable us to *avoid* the causes of inflammation of the nipple; but by their use, we may render the operation of them less mischievous, which is often sufficient in favorable cases to effect a cure. In considering the curative remedies for sore nipples, I must protest against the simplicity with which we use the word, and think of sore nipples. We speak and think of it as though there was no variety of sore nipples. The same treatment is not applicable to abrasions, that is to chaps or cracks, nor to ulceration, nor to all the conditions of ulceration. Nature tries to cure cuticular abrasions by an effusion upon the naked surface of a viscid albuminous layer, thus defending the delicate tissue from contact with atmospheric air, or other irritating substances, and if this is allowed to remain undisturbed, it will, as it falls from the place, leave a well-formed delicate cuticle. And I think the nearer we imitate nature in this respect, the more good we will do. We may use starch or mucilage to cover the abrasions, but any astringent or stimulant application is inadmissible. Abrasions, however, do not last long without becoming ulceration, and the treatment may be different. When there are numerous fine chaps covering a large surface of the nipple, or when single, if very shallow, the treatment for abrasions will usually answer every purpose.—Ointments of a mild unirritating, or even a soothing quality, are probably more applicable than in abrasions. The following is a very good one:

℞ Cerat. Alb. ℥ ii.

Ol. Amyg. Dulc, ʒ i.

Mel. Desp. ℥ ss.

Mix. Dissolve with gentle heat, and add Bals. Canad ʒ iiss,

This should be applied every time after nursing. When the cracks are deep, it is indispensable to quick cure that they should be closed up, and kept so until complete adhesion of their sides takes place. This may usually be done with great facility in the following manner, viz: Press the nipple in such a way as to close the crack, and while thus holding it, apply a thick layer of collodion over the surface. We should apply the layer thickly, and have it extend some distance in every direction, so that it will keep the crack together. The collodion is not easily sucked off by the child, and if the nipple shield be used, it need not be disturbed at all until completely healed. We should watch the coat of collodion, and remove it when it seems to be becoming deficient by violence of nursing. In most cases this covering, if kept up inviolable for a week, will suffice to complete a cure if suppuration is not going on in the chapped place. If this is the case, and the surface becomes an ulcerated one, it will fill up by granulation alone, and falls into the catagory of ulcerations. In this part of the body, ulceration does not differ from the conditions it assumes in other places, and it cannot be expected that I should dwell upon every variety that may occur. General principles must guide us here as elsewhere. There are two conditions, however, one of which is apt to obtain a prominence and give character to this ulcer, acute and chronic; in either of these conditions the ulcer may be exceedingly irritable to touch, and painful, and in the latter, indolent and atonic. The acute variety is apt to be attended with considerable heat, tumefaction, color and tenderness. These conditions should be removed by depletion, as by leeches, one or two will generally do, cold emollient poultices, large enough for the nipple alone, and removed as often as they become warm. Or we may envelope the nipple in a thin layer of thick mucilage, covered by oil silk, so as neatly to fit the organ, kept cold by ice applied in a minute bladder or india rubber bag, or we may wrap the ice in oil silk.—In whatever envelope it is used, it should not extend beyond the inflamed part, and should be separated from it by a thin layer of cotton wool, or something of that kind.

When such remedies are not necessary because of the non-existence of these symptoms, we should content ourselves in the very early stages of ulceration with similar mucilagenous and bland ointment applications as in abrasions, but as the process goes on, and the acute symptoms entirely subside, astringents become useful, and these will vary in character and strength according to indications of atony and flabbiness, &c. Alum and tannin are excellent applications at first, but will have very little effect after it has continued for any great length of time. Sulphate of zinc and borax will come next in respect to time. One scruple of tannin to one ounce of rose water, five grains of alum, the same quantity of sulph. zinc, are all good in the earliest stages of ulceration of the nipples, when the more acute symptoms have subsided. The following formulæ are often very useful.

℞ Glycerine, ʒ ii.

Soda Subboras, ʒ ss.

Aquae Rosae, ℥ iss.

Mix. Use as a wash each time after sucking. Or,

℞ Soda Subboras, ʒ ii.

Cretae prep, ℥ j.

Spts. Vini,

Aquae Rosae, aa. ℥ iij.

Mix and dissolve. This last may be used when the ulcer is becoming somewhat indolent. Tinct. Kino, Tinct. Nut Galls, and in fact, almost every astringent has been used in these ulcers. In chronic ulcers, still stronger astringents or stimulants will become necessary in conjunction with other remedies. A skillful use of the Suph. Cupri, and Nit. Argent, will do a great deal to heal up and shorten the course of these chronic ulcers. The Nitrate has done the most good in my hands. It should be applied in substance to the surface of the ulcer, and never be used oftener than once in eight days, when a second application becomes necessary. Between times, the ulcer may be dressed with some of the milder astringents, alum or tannin, for instance, in solution In the irritable variety, some narcotic extract should be made into ointment: Belladonna, hyosciamus, Opium, &c. An excellent expedient, and one that will often entirely change the character of these ulcers, is to anæsthetize the part with ice, as is directed to be done on a part before the performance of an operation.

We are very apt after we begin to use curative measures, to neglect the palliation. This is a great mistake, for they can have but little good influence, while the causes are allowed to act with all the power that is necessary to produce the disease. We cannot attach too much importance to the measures of palliation.

---

*Treatment of Inflammation of Lymphatic Glands.*—The causes of lymphatic inflammation should receive our attention first, as the abraided or ulcerated nipples, inflamed areola or integuments of the breast, or when chronic, the constitutional condition in addition to the local excitements. When acute, they will require in addition, the antiphlogistic measures adopted in other inflammations, leeches, cooling lotions, fomentations, cathartics, &c. When chronic, alteratives, iodine tonics, liniments, irritants, &c., which will be adapted, by every physician according to his own judgement, to the peculiarities of his case. If we are accurate in our diagnosis, and seperate this affection from those of the deeper seated structures, there will be no great difficulty in adjusting the treatment of it.

The treatment of milk abscess, is one, however, of greater importance, because of its frequent occurance and distructive effects. The remedies naturally range themselves into preventive and curative. The prevention has reference to the management of the anatomical and pathological conditions of the nipple, which prevents the free elimination of the milk. Of the latter, I have written quite as extensively as the limits of this paper will allow. Can we change the anatomical deficiencies or depraved shape of the nipple, of congenital or acquired origin? It is a matter of the utmost importance to the health and happiness of the patient, that this question should be decided promptly and properly. Much will depend upon, whether our attention was drawn to the case early in pregnancy, or not until the time of labor, or even afterwards, as to the probability of success in many cases. In other cases, we can decide the nipple to be impracticable from the first sight, at whatever time we examine it, and I would insist upon the impropriety of compelling a woman to pass through the terrible pain and exhaustion, which attend these cases where the nipple, for instance, is entirely wanting, and prehension impossible, as represented in fig. 1. If our attention be not drawn to the nipple until after labor, and the functions of the breast are required, we ought not to hesitate to decide against nursing, or attempting it. And so far as I am concerned, individually, I would advise against the endeavors to use the breast, represented by fig. 1, if I was aware of its conditions at the beginning of pregnancy. Fortunately this deficiency is rare. When there is an approximation to this, but not complete absence or depression of nipple, the breasts shaped like figs. 2 and 3, much may be done toward rendering them useful, provided our efforts are judicious, and sufficiently prolonged. They should be commenced as soon as pregnancy is known to have taken place; and if in the state of society it were practicable, the prospect of success would be much better, could we have the management of our patients as soon as menstruation began. If mothers were well instructed in such matters, and would carefully attend to it, the probability is, that almost no cases of anatomical unfitness for nursing, would present themselves.

Nipples, represented in Nos. 2, 3 and 4, if not observed by the practitioner, until after parturition, will be almost certain to give us trouble, and in 2 and 3, we will be scarcely able to prevent extensive milk abscesses. The first, and most important principle, is to take perpendicular pressure entirely off the top of the nipple, and this would probably be sufficient to prevent the difficulty, if complete. This little projection on account of the fashions of female dress, is kept constantly pressed back into the soft yielding mammary tissues, until it becomes hopelessly imbedded into them. Now, what we want, is to counteract, and remedy the effect of this mischievous habit. Quite a number of devices have been resorted to, for the purpose of starting the nipple forward from its embeded condition. They have for their object, as a general thing, the production of counter pressure around the nipples, upon the areola, and central portion of the breast, in such manner as to press the central tissues beneath the nipple, and thus cause it to protrude. If this object can be effected by such gentle means, continued for a sufficient length of time before the birth of the child, as to make it a permanent state of this organ, the treatment will be effected. The misfortune is, we can seldom get the important desideratum (time,) and we are under the necessity of beginning our treatment, often too late to effect anything. When called upon to remodel a nipple before, or during pregnancy, we may make use of a shield of stiff silver, or iron wire, large enough to embrace, and actually pit the anterior surface of the breast, with a cap-like projection from its center, into which the nipple may project. There may be some soft substance, very thin cotton or wool, to protect the surface from the wire placed immediately beneath it. This should be worn for months under the dress, and receive all the pressure from it, and distribute it over the front of the mamma, and protect the nipple from any pressure. Such a shield is far better than ivory, wood, india rubber, or any other impermeable substance, as it does not interfere with the transpiratory functions of the skin, or the secretion of the areolar follicles, and glandula.

When we are not called upon to treat these rudimentary nipples, until the time of, or after parturition, such treatment will not avail.

The effect must be brought about more promptly, on account of the necessity for immediate use. In many cases the nipple can be made available by temporarily inducing its erection by simple titillation with the finger, moving it gently around it, and then immediately applying the child. An excellent way of erecting the nipple, when there is considerable depression, is to place a thick layer of collodion around it on the areola. When this dries and contracts, the nipple will be elevated quite prominently. The child should then be placed to the breast, and allowed to nurse.

When the nipple is protruded in some of these ways, the milk may usually be drawn, so as to, more or less, completely empty the reservoirs. This will prevent milk abscess, very generally. When inflammation of the reservoirs has fairly begun, it will be exceedingly difficult to prevent suppuration. The curative means consist in thoroughly evacuating and keeping empty this set of vessels. Several modes of doing this, have been recommended—such as drawing with a glass tube, shaped like a pipe. Various shapes of breast tubes, and pumps are in use, but I must object to *all* of *these.* It is a very easy matter to injure the delicate tissues of the breasts, by the hard rim of these instruments, and I think the accident often happens.

A puppy is often brought into requisition for this purpose, but is rough, and sometimes irritates the nipple and even sucks the skin off it. The only proper thing for drawing the milk is the mouth, and when these reservoirs are inflamed, it should be the mouth of an adult, who can vary the pressure or force to suit the tenderness of the part. Another very useful class of measures are those intended to suppress the secretion of the milk, and thus relieve the reservoirs from the distension. The narcotic substances taken internally or applied externally to the breast, do a great deal towards stopping the secretion of the milk. Opium in large doses, so as to keep the patient very thoroughly under its influence, aids very much in arresting the secretion of milk. Applied externally in ointments, so as to produce a decided impression upon the system, has a similar effect; but belladonna seems to have acquired most renown for this purpose. Mr. Richard Marley reported forty cases to the Obstetrical Society of London, treated successfully by inunction of belladonna. Dr. Tanner corroborates Mr. Marley's conclusions by his own experience. American physicians testify in favor of belladonna, by furnishing to medical periodicals a great many favorable cases within the last two or three years. It should be remembered that many of the extracts sold in the shops, if not entirely inert, are at least much below the standard in strength. Our opinion of the efficacy of these, of course, will vary from this circumstance, and hence, doubtless the discrepancy in the testimony of different observers in regard to the use of belladonna, for the purpose of suppressing the secretion of milk. The inunction of ointment made with the extract, should be carried to a sufficient extent to produce some of its characteristic effects upon the system. Its use should be as profuse as the system will well bear. Cold, as a local application in cases of milk abscess, has several good effects. It anæsthetizes the part, rendering the patient more comfortable, it decreases the secretion, constringes the reservoirs of milk, and allays excitement in the capillary circulation. In supplying cold to the breast, the temperature should be about forty or forty-five degrees, and kept as steadily at that as possible. Water of that temperature might be kept running through an india rubber bag enveloping the organ. A bladder partly filled with ice and water, with a piece of flannel between it and the skin, would also do very well. When we do not desire to promote secretion of milk, cold may be used. I do not believe there is any danger from it while its application is confined to the part affected, and its bad effects are usually produced by wetting the clothing, or allowing it to get applied to other parts of the person. I cannot express with sufficient force, the evil effects which the prejudices of a former age in medicine, have fastened upon the minds of at least a part of the public, in the practice of keeping the breasts wrapped in thick layers of cotton or lamb's wool. It is promotive of the secretion of milk by drawing blood to the gland, and thus keeps up the state of things we desire to avoid. For internal treatment, a saline cathartic every other day, and two grains of Iodide of Potassium every four hours, may be relied upon as materially aiding the other treatment. In this affection, antiphlogistic treatment is merely auxillary, and should not be pushed to an extent usually considered necessary in other inflammatory affections. In this case, over distension is the cause of the inflammation, and its removal in the early stages is generally sufficient to cure.

Acute inflammation of the glands of the breast, when it occurs as the effect of congestion immediately preceding the secretion of milk, is apt to be very extensive, sometimes involving the whole of the gland, and will require energetic treatment. For the first few hours, we should try warm fomentations with the hope of establishing the secretion. This probably would be unavailing if actual inflammation had begun; but we cannot always determine the point when this intense congestion passes into inflammation, and hence we are justified, I think, in making the effort. If the patient is robust, and the fomentations fail wholly or partially to bring relief, a decided venesection will often turn the balance in favor of resolution. When we bleed, the object should be to produce a decided impression; and in order to do this, the patient should be in a sitting posture, and the blood allowed to run until the pulse is affected and syncope approaches. I have so much faith in verat. viride in combating inflammation, that I should begin its use immediately after v. s., and if the patient is strong, give it in six drop doses every four hours until the pulse is brought down to sixty in the minute, and then by administering it in decreased doses, keep it as nearly at that as possible. One grain of calomel with a quarter of a grain of sulph. morph., may be given occasionally, if the pain is urgent, every four or six hours. This kind of promptitude and energy of treatment, will frequently arrest the inflammation and bring about resolution. And when we remember the amount of suffering and damage it may prevent, nothing should deter us at least from urging our patient to accept the treatment.—Should this not be sufficient, it is an important question whether depletion can be carried further. One good full general bleeding, if followed by veratrum, will be sufficient generally; but sometimes it will be expedient to use leeches, and produce a general alterative mercurial influence. A lotion made of one part of sulph. ether to two parts of alcohol, will be a good soothing adjunct after the inflammation becomes permanent. If the inflammation begins later, the extent of disease is apt to be less, and may be confined to one lobule, or at most, a part of the gland only. In this case, a brisk cathartic of calomel, aided by some saline, leeches to the part, followed by cold lotions, tinct. verat. viride, or solution of tart. ant., given at sufficient intervals, in proper quantities, will afford us efficient treatment. If this treatment is begun early, we may expect much good from it. It has always been an interesting question with me, after the inflammation has existed for a length of time, and we cannot avoid the formation of pus, whether we should abandon antiphlogistic means and resort to warm poultices and fomentations to promote suppuration. I think that this is not justifiable in many instances. The probability is, that if we continue our general and local antiphlogistic treatment until suppuration is clearly evident, we may limit the extent of that termination, lead to resolution in a larger part of the gland than would otherwise take place, and thus save much of the glandular tissue. When the *whole gland* is inflamed, there is no necessity, in fact I think it injurious to institute and continue strenuous efforts to draw the breast. There is little or no secretion, and when a part of the gland only is inflamed, and milk is produced by the rest of it, it is questionable whether anything but the most moderate means for this purpose are admissible. Retained milk is not the cause of inflammation in this case as in milk abscess. Very frequently glandular inflammation is complicated with inflammation of the reservoirs; then we must combine our treatment to suit the case, local and general antiphlogistic, with means to arrest the secretion and empty the reservoirs of the milk already contained in them.—Chronic inflammation of the gland will be cured by much the same treatment successful in other glandular inflammations of this grade; leeches, mercurials, iodine and vegetable alteratives perseveringly administered internally, and locally applied. Much reliance can be placed upon well regulated and graduated pressure, with adhesive straps pressing the part diseased, against the ribs; or collodion encasing the breast thoroughly. When suppuration has taken place, what are the indications to be relied upon to justify us in evacuating it? There can be no doubt, I think, that the earlier the matter is let out the better for several reasons. The cavity becomes larger by allowing it to remain, it burrows through the surrounding tissues; the longer it remains, the greater the amount and duration of the irritative fever that accompanies its retention. But notwithstanding the desirableness of getting rid of the pus, we should hesitate to cut through uncondensed tissue to any extent. In cases where the inflammation and suppuration are deep in the gland, it is desirable to wait until the pressure from within has lasted long enough, and in a sufficient degree to cause the condensation of the tissue. Otherwise, it will require a very large opening to allow a free discharge. I think we should not lance the part until fluctuation is quite evident, and the pus has made its way to the fascia or integuments. It is never desirable to cut through any part of the uninjured gland or milk ducts, and altogether, I should feel more inclined to allow it to approach the integuments very closely before cutting.

In the case of milk abscess, the earlier the opening is made, the better. As soon at it is evident that suppuration is inevitable, the opening may be made. The smaller the opening, to allow the escape, the better. Should the disease still exist that caused the retention, the opening should be preserved. Often the evacuation of one or two reservoirs will suffice, and the rest will continue to discharge through the nipple. The effect of suppuration, and evacuation of a milk reservoir, is often to entirely destroy its cavity, but in other instances, it continues to discharge through the artificial opening, and a milk fistula remains. This may be closed by an occasional application of the nitrate of silver in pencil. Worse than these are the tortuous lacuna, that sometimes result from the deep glandular abscess of the breast. They are generally difficult to cure. Injection of iodine, is the remedy most relied upon for these troublesome sequences to suppuration. The most effective way to inject. is to insert a soft flexible catheter, if possible, to the bottom of the twisted canal, and throw the injection through it, so as to apply it without dilution to the bottom of the pus fistula. I think this important, when practicable, because it favors the shallowing, instead of the narrowing of the cavity.

Of course it is never advisable to slit up these obstinate puriferous ducts in the breast, as it sometimes is in other parts of the body, because, of the amount of tissue that might be damaged, which it is desirable to save.

## Figures and Tables

**Fig. 1. f1:**
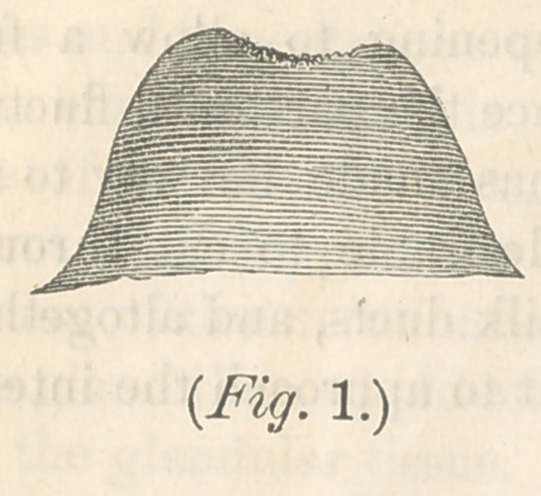


**Fig. 2. f2:**
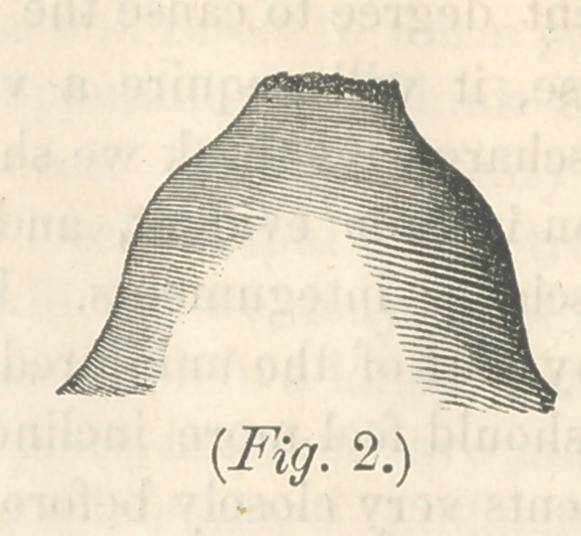


**Fig. 3. f3:**
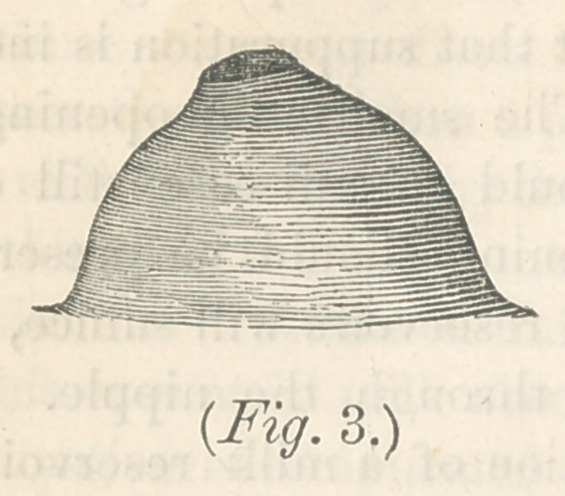


**Fig. 4. f4:**
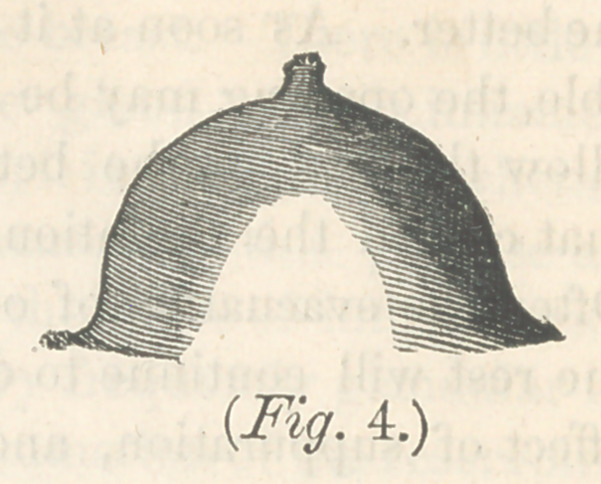


**Fig. 5. f5:**
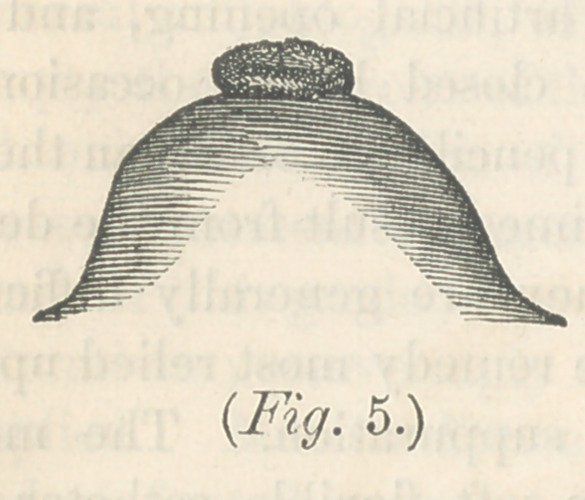


**Fig. 6. f6:**
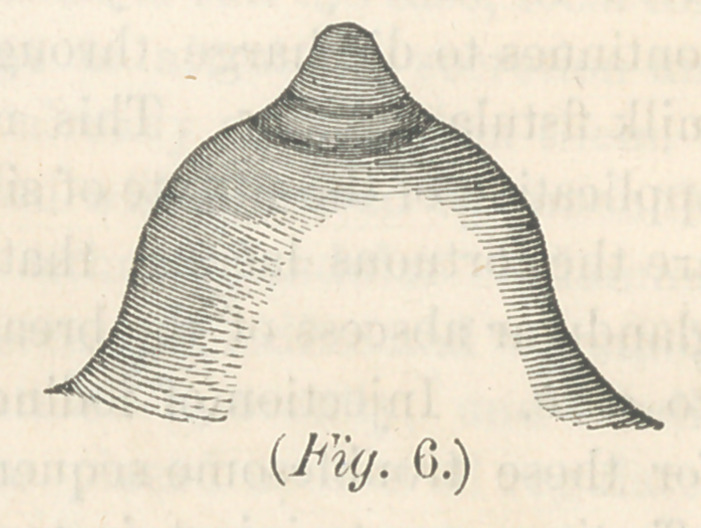


**Fig. 6. f7:**
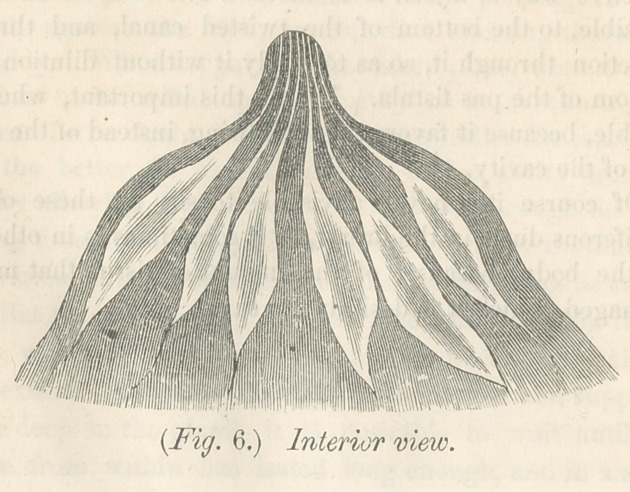


**Fig. 1. f8:**
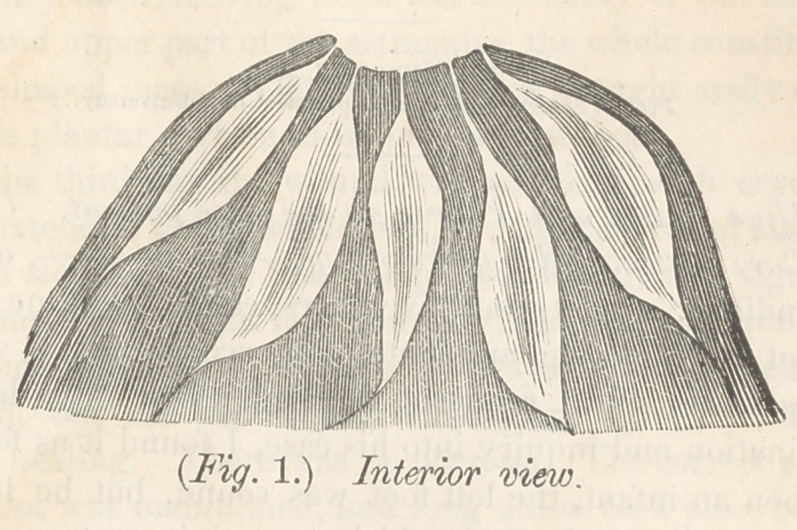


**Fig. 5. f9:**